# A malignancy risk prediction model for Bethesda category III thyroid nodules based on serological indicators, C-TIRADS, and BRAF V600E testing

**DOI:** 10.3389/fendo.2026.1919450

**Published:** 2026-09-24

**Authors:** Wei Yang, Chun Jin Xiong, Shuang Shuang Dong, Bin Sheng

**Affiliations:** 1 Department of Medical Pathology,Northern Jiangsu People's Hospital Affiliated to Yangzhou University, Yangzhou, China; 2 Department of Ultrasound,Northern Jiangsu People's Hospital Affiliated to Yangzhou University, Yangzhou, China; 3 Department of Medical Laboratory,Northern Jiangsu People's Hospital Affiliated to Yangzhou University, Yangzhou, China

**Keywords:** Bethesda category III, BRAF V600E, C-TIRADS, multimodal model, TgAb, thyroid nodules, TPOAb

## Abstract

**Objective:**

To investigate the diagnostic value of a multimodal prediction model integrating thyroglobulin antibody (TgAb), thyroid peroxidase antibody (TPOAb), the Chinese Thyroid Imaging Reporting and Data System (C-TIRADS), and BRAF V600E mutation status, for evaluating the malignancy risk in Bethesda Category III thyroid nodules.

**Methods:**

We retrospectively enrolled 93 patients whose thyroid nodules were diagnosed as Bethesda Category III via fine-needle aspiration and subsequently underwent surgical treatment at Northern Jiangsu People’s Hospital between July 2020 and June 2025. Preoperative TgAb levels, TPOAb levels, C-TIRADS classification, and BRAF V600E mutation status data were collected. A malignancy risk prediction model was constructed using Firth’s penalized likelihood logistic regression. The diagnostic performance of individual indicators and the combined model was evaluated using receiver operating characteristic (ROC) curves. A nomogram was developed for individualized risk prediction, and the model was internally validated using bootstrap resampling (1000 iterations), calibration curves, and decision curve analysis (DCA) to assess the clinical utility.

**Results:**

Among the 93 Bethesda III thyroid nodules, 84 (90.32%) were malignant. Among the individual predictors, BRAF V600E exhibited the highest diagnostic performance (AUC = 0.736, 95% CI: 0.589–0.884). Compared with individual modalities, the combined multimodal model demonstrated a significantly improved diagnostic trend, with an AUC of 0.821 (95% CI: 0.653–0.989).

**Conclusion:**

In a surgically enriched, high-risk cohort of patients with Bethesda III thyroid nodules, the multimodal model integrating TgAb, TPOAb, C-TIRADS, and BRAF V600E testing provides excellent auxiliary diagnostic value and can effectively guide preoperative risk stratification.

## 1 Introduction

Thyroid nodules are common endocrine disorders, and their detection rates have steadily increased with the widespread application of high-resolution ultrasonography (1). Fine-needle aspiration (FNA) cytology is currently the standard method for preoperative evaluation of benign and malignant thyroid nodules (2). However, Bethesda Category III nodules—defined as atypia of undetermined significance or follicular lesion of undetermined significance (AUS/FLUS)—represent a diagnostic “gray zone” because of their cytological heterogeneity and fluctuating malignancy risk (3). According to the Bethesda System for Reporting Thyroid Cytopathology (2017), the estimated malignancy risk for Bethesda III nodules ranges from 5% to 30% (4). Because of this profound diagnostic uncertainty, clinical management strategies remain controversial. Over-reliance on surgical intervention may subject patients with benign nodules to unnecessary operations, while conservative follow-up may delay the timely diagnosis and treatment of malignant lesions. Therefore, establishing a more precise risk stratification system is critically important for the clinical management of Bethesda III nodules.

In recent years, ultrasound-based risk stratification systems have been increasingly utilized in the diagnosis of thyroid nodules (5). The Chinese Thyroid Imaging Reporting and Data System (C-TIRADS) standardizes the evaluation of malignant sonographic features—such as hypoechogenicity, microcalcifications, and irregular margins—thereby improving the detection rate of malignant thyroid nodules (6). However, ultrasound evaluation is inherently constrained by operator dependency and has limited diagnostic capacity to detect certain follicular neoplasms and low-invasive carcinomas (7, 8).

Recent molecular classifiers, such as Afirma GSC and ThyroSeq v3, have improved the risk stratification of indeterminate thyroid nodules but has limitations such as cost and accessibility (9, 10). Therefore, practical models based on readily available markers remain clinically valuable. BRAF V600E, despite its limited sensitivity, remains an attractive biomarker because of its high specificity, availability, and potential integration with clinical and ultrasound features (11). Despite its utility, the sensitivity of the BRAF V600E mutation alone is insufficient for Bethesda III nodules, as some malignant cases may be driven by alternative molecular events, such as RAS mutations or RET/PTC fusions (12, 13). Consequently, relying solely on BRAF V600E testing fails to fully meet clinical demands.

An increasing body of evidence suggests that immune-related serological markers, such as thyroglobulin antibody (TgAb) and thyroid peroxidase antibody (TPOAb), may be associated with the occurrence and progression of thyroid cancer, as well as alterations in the tumor immune microenvironment (14). Nevertheless, research evaluating the combined application of serological immunological indicators, ultrasonography, and molecular testing for risk stratification in Bethesda III nodules remains scarce.

These parameters integrate imaging, molecular, and immunological information, capturing the biological characteristics of thyroid nodules from multiple dimensions. This study aims to construct a malignancy risk prediction model for Bethesda III thyroid nodules by integrating serological indicators, C-TIRADS classifications, and BRAF V600E mutation status. By evaluating the clinical diagnostic value of this multimodal approach, this study provides a robust reference framework for the precise diagnosis and treatment of Bethesda III nodules.

## 2 Materials and methods

This retrospective cohort study recruited consecutive patients who underwent ultrasound-guided fine-needle aspiration (FNA) for Bethesda III thyroid nodules at Northern Jiangsu People’s Hospital between July 2020 and June 2025. Clinical, ultrasound, serological, and molecular data were retrospectively collected and analyzed.

### 2.1 Patients and study design

The study population consisted of patients with Bethesda Category III thyroid nodules who underwent ultrasound-guided FNA during the study period. The sample size was determined by the number of eligible patients available during the predefined study period. All patients who met the predefined inclusion and exclusion criteria were consecutively included. No *a priori* sample size calculation was performed because of the retrospective design, and no additional sampling or selective case inclusion was performed to minimize selection bias. All the nodules were assessed by ultrasound; however, only the nodules that underwent fine-needle aspiration (FNA) and subsequently yielded a Bethesda category III cytological diagnosis were included in the analysis. Other nodules from the same patient were excluded from the study to ensure that each case corresponded to a single index nodule.

The inclusion criteria were: (1) the nodules were cytologically diagnosed as Bethesda Category III; (2) patients underwent preoperative serological testing for TgAb and TPOAb, C-TIRADS ultrasound classification, and BRAF V600E gene mutation testing; and (3) patients subsequently underwent surgical resection and had a definitive postoperative histopathological finding.

The exclusion criteria were: (1) ambiguous or missing preoperative testing results; (2) a history of preoperative anti-tumor therapies, such as radiotherapy, chemotherapy, or immunotherapy; or the presence of multiple postoperative pathological nodules where the biopsied nodule could not be definitively matched to the surgically resected nodule; (3) the presence of other malignancies; (4) the presence of coexisting thyroid disorders such as hyperthyroidism, hypothyroidism, or Hashimoto’s thyroiditis; and (5) a prior history of thyroid surgery.

### 2.2 Clinical measurements and data collection

#### 2.2.1 Serological evaluation of TgAb and TPOAb

A 3 ml sample of fasting venous blood was drawn from each patient in the morning. The samples were centrifuged at 3000 r/min for serum separation prior to testing. Serum levels of TgAb and TPOAb were quantified utilizing a chemiluminescence immunoassay. Measurements were performed using a Cobas e801 fully automated chemiluminescence analyzer, with reagents and quality control sera provided by Roche Diagnostics (Germany). The positivity thresholds were defined as a TgAb concentration > 100 IU/ml and a TPOAb concentration > 50 IU/ml. Blood samples were collected from all patients after an overnight fast of at least 8 hours. Venous blood samples were collected from the antecubital vein, at the clinical laboratory of Northern Jiangsu People’s Hospital before ultrasound-guided FNA. Serum samples were separated by centrifugation and stored for subsequent analysis of TgAb, TPOAb, and other biochemical parameters.

#### 2.2.2 C-TIRADS ultrasound classification

Ultrasound examinations were independently reviewed by two radiologists with 10 and 8 years of experience in thyroid examination in accordance with the Chinese Thyroid Imaging Reporting and Data System (C-TIRADS) guidelines (5). The radiologists were blinded to the clinical information, serological results, molecular testing results, and final postoperative histopathological findings. Positive malignant sonographic indicators included (1) a vertical orientation (taller-than-wide shape), (2) a solid composition, (3) marked hypoechogenicity, (4) punctate echogenic foci (suspected microcalcifications), and (5) blurred/irregular margins or extrathyroidal extension. A benign indicator was defined as a punctate echogenic focus with comet-tail artifacts. The ultrasound malignancy risk score was calculated by assigning 1 point for each positive malignant feature; if a benign comet-tail artifact was present, 1 point was subtracted. In cases of disagreement, a consensus was reached through discussion. Inter-observer agreement for C-TIRADS classification between the two radiologists was assessed using weighted Cohen’s kappa coefficient (**Supplementary Figure 1**).

To optimize the specificity of the multimodal model for screening high-risk nodules, a strict cutoff value was used: nodules classified as C-TIRADS 4B and below were categorized as benign, whereas those classified as C-TIRADS 4C and above were categorized as malignant.

#### 2.2.3 Fine-needle aspiration and BRAF V600E mutation analysis

Ultrasound-guided FNA was performed on the thyroid nodules, and two separate aspirates were collected. One aspirate was prepared for cytological examination and classified according to the Bethesda System for Reporting Thyroid Cytopathology, whereas the other was used for molecular analysis. Genomic DNA was extracted from the FNA specimens using a commercial DNA extraction kit according to the manufacturer’s instructions. The extracted DNA was stored at −20 °C until further analysis.

The BRAF V600E mutation was detected using a BRAF Gene Mutation Detection Kit (PCR-fluorescence probe method; Beijing Xinnuo Meidi Gene Detection Technology Co., Ltd., Beijing, China). Real-time PCR amplification and fluorescence signal acquisition were performed using an ABI real-time PCR system (Applied Biosystems, Foster City, CA, USA) according to the manufacturer’s protocol. Mutation status was determined on the basis of cycle threshold (Ct) values. Samples with a Ct value <28 were considered BRAF V600E mutation-positive, whereas samples with a Ct value ≥28 were considered BRAF V600E mutation-negative. Positive and negative controls were included in each assay run to ensure detection reliability.

#### 2.2.4 Postoperative histopathological findings

Patients with Bethesda category III thyroid nodules who underwent surgical resection were included in this retrospective cohort study. The decision to perform surgery was made through a multidisciplinary clinical evaluation on the basis of the cytological diagnosis, ultrasound risk stratification, molecular testing results, nodule characteristics, and patient preference. The main indications for surgical resection included (1) persistent diagnostic uncertainty associated with Bethesda category III cytology and the need for definitive postoperative histopathological findings; (2) suspicious ultrasound features suggestive of malignancy according to the C-TIRADS classification; (3) positive molecular findings, particularly the presence of the BRAF V600E mutation, indicating an increased risk of malignancy; (4) significant nodule growth or other concerning changes during clinical follow-up; and (5) symptomatic nodules causing local compression or other clinical concerns.

Following surgery, the resected thyroid specimens were fixed in 10% neutral buffered formalin, routinely processed, paraffin-embedded, and stained with hematoxylin and eosin (H&E). Postoperative histopathological findings were independently reviewed and confirmed by experienced pathologists according to the World Health Organization (WHO) Classification of Tumors of Endocrine Organs. Thyroid nodules were subsequently classified as malignant or benign on the basis of the final postoperative histopathological findings, which served as the definitive reference standard for evaluating the diagnostic performance of the predictive models.

### 2.3 Statistical analysis

All the statistical analyses were performed using SPSS 26.0 and R software version 4.3.1. Categorical variables are presented as frequencies and percentages [n (%)].

Using postoperative histopathology as the gold standard, the diagnostic performance of the TgAb, TPOAb, and C-TIRADS classifications and the BRAF V600E mutation status was individually evaluated. To construct predictive models, the clinical outcome (nodule status) was coded as ‘1’ for malignant tumors and ‘0’ for non-malignant cases. Given the pronounced imbalance between malignant and benign outcomes in this surgical cohort, standard maximum likelihood estimation was susceptible to complete separation and parameter bias. Therefore, TgAb, TPOAb, C-TIRADS classification, and BRAF V600E status were incorporated into a Firth penalized likelihood logistic regression model to construct the combined prediction framework. Odds ratios (ORs) and their corresponding 95% confidence intervals (95% CIs) were calculated.

The diagnostic efficacy of individual markers and the combined model were evaluated using Receiver Operating Characteristic (ROC) curves, with performance quantified by the area under the curve (AUC) and 95% CI. A nomogram based on the Firth logistic regression results was constructed to provide individualized visual predictions of malignancy risk.

Internal validation of the model was performed using bootstrap resampling (1000 iterations), and a calibration curve was plotted to evaluate the concordance between the predicted probabilities and actual observed outcomes. Furthermore, Decision Curve Analysis (DCA) was performed to assess the net clinical benefit and practical application value of the predictive models across various threshold probabilities. A two-sided P value < 0.05 was considered to indicate statistical significance.

### 2.4 Equipment and laboratory measurements

Thyroid ultrasound examinations were performed using a LOGIQ E10 ultrasound system (GE Healthcare, Chicago, IL, USA) equipped with a high-frequency linear-array transducer. Ultrasound-guided fine-needle aspiration (FNA) was performed using disposable aspiration needles (BD Biosciences, Franklin Lakes, NJ, USA). Ultrasound findings were evaluated according to the Chinese Thyroid Imaging Reporting and Data System (C-TIRADS) criteria.

Peripheral venous blood samples were collected after overnight fasting at the Department of Laboratory Medicine, Northern Jiangsu People’s Hospital. Serum thyroglobulin antibody (TgAb) and thyroid peroxidase antibody (TPOAb) levels were measured using an automated electrochemiluminescence immunoassay analyzer (Cobas e801, Roche Diagnostics, Mannheim, Germany) with corresponding reagent kits (Roche Diagnostics, Mannheim, Germany) according to the manufacturer’s instructions.

BRAF V600E mutation detection was performed using a real-time PCR-based mutation detection kit (Beijing Xinnuo Meidi Gene Technology Co., Ltd., Beijing, China) according to the manufacturer’s instructions. PCR amplification and fluorescence signal detection were performed using an Applied Biosystems 7500 Real-Time PCR System (Applied Biosystems, Foster City, CA, USA).

Statistical analyses were performed using SPSS software (version 26.0; IBM Corp., Armonk, NY, USA) and R software (version 4.3.1; R Foundation for Statistical Computing, Vienna, Austria). Receiver operating characteristic (ROC) curve analysis and DeLong tests were performed using the relevant R packages.

## 3 Results

### 3.1 Patient characteristics

A total of 93 patients who met the inclusion criteria between July 2020 and June 2025 were enrolled in this study. The cohort comprised 11 males and 82 females, with the age ranging from 22 to 76 years (median age: 48.5 years). Following surgical resection, a total of 93 nodules were evaluated pathologically. Postoperative histopathological findings confirmed that 84 nodules (90.32%) were malignant, all of which were papillary thyroid carcinomas, whereas 9 nodules (9.68%) were benign, consisting entirely of follicular nodular disease (**Figure 1**).

**Figure 1:**
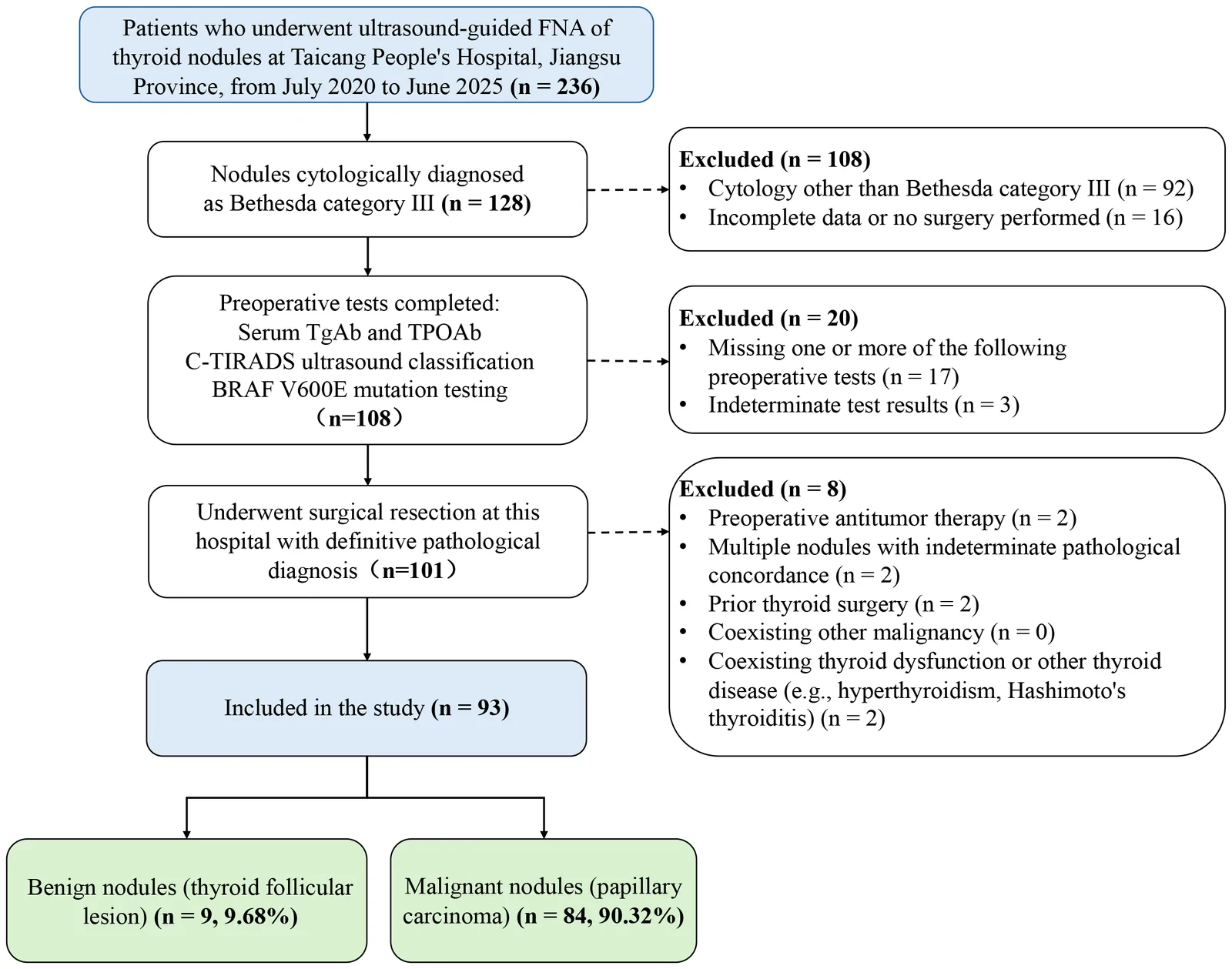
Flowchart illustrating the patient selection and allocation process.

### 3.2 Comparison of individual indicators against postoperative histopathological findings

Among the 93 Bethesda III nodules, the incidence of malignancy and benign disease was 90.32% (84/93) and 9.68% (9/93), respectively. The diagnostic distributions of TgAb, TPOAb, C-TIRADS, and BRAF V600E relative to the final pathological outcomes are presented in **Table 1**.

**Table 1:** Comparison of the diagnostic results of TgAb, TPOAb, C-TIRADS, and *BRAF* V600E testing with postoperative histopathological findings in Bethesda category III thyroid nodules.

Variables	Overall N = 93^1^	Benign N = 9^1^	Malignant N = 84^1^
**TgAb**	18	1	17
**TPOAb**	21	1	20
**C-TIRADS**			
1	53	8	45
2	20	0	20
3	6	1	5
4	8	0	8
5	6	0	6
**BRAF V600E**	50	1	49
^1^ n			

### 3.3 Firth logistic regression analysis and forest plot

To identify independent influencing factors for malignancy in Bethesda III nodules, a multivariable analysis of TgAb, TPOAb, C-TIRADS classification, and BRAF V600E mutation status was conducted utilizing the Firth penalized likelihood logistic regression model. The results indicated that the BRAF V600E mutation was significantly associated with nodule malignancy and was an independent risk factor (OR = 9.027, 95% CI: 1.823–89.446; P = 0.005). Patients harboring a positive BRAF V600E mutation had an approximately 9-fold increased risk of developing thyroid cancer compared with those with negative results (wild-type). Conversely, while TgAb (OR = 1.852; 95% CI: 0.150–36.071; P = 0.645), TPOAb (OR = 1.289; 95% CI: 0.101–25.894; P = 0.852), and C-TIRADS classification (OR = 1.588; 95% CI: 0.804–6.625; P = 0.219) demonstrated a trend toward increased risk, the difference was not statistically significant (all P > 0.05) (**Table 2**).

**Table 2:** Multivariable firth logistic regression analysis of risk factors for malignancy in Bethesda category III thyroid nodules.

Variable	OR	Lower_CI	Upper_CI	P_value
TgAb	1.852	0.150	36.071	0.645
TPOAb	1.289	0.101	25.894	0.852
C-TIRADS	1.588	0.804	6.625	0.219
BRAF V600E	9.027	1.823	89.446	0.005

The forest plot provided a visual representation of the magnitude of the impact of each variable on malignancy risk (**Figure 2**). The BRAF V600E mutation had the highest OR, with its entire 95% confidence interval situated to the right of the reference line (OR = 1), affirming a stable and significant positive correlation with malignant outcomes. The 95% confidence intervals for the TgAb, TPOAb, and C-TIRADS classifications intersected the OR = 1 reference line, indicating their limited independent predictive capacity. Overall, the BRAF V600E mutation emerged as the most valuable predictive risk factor and is capable of providing critical support for risk stratification and clinical decision-making.

**Figure 2:**
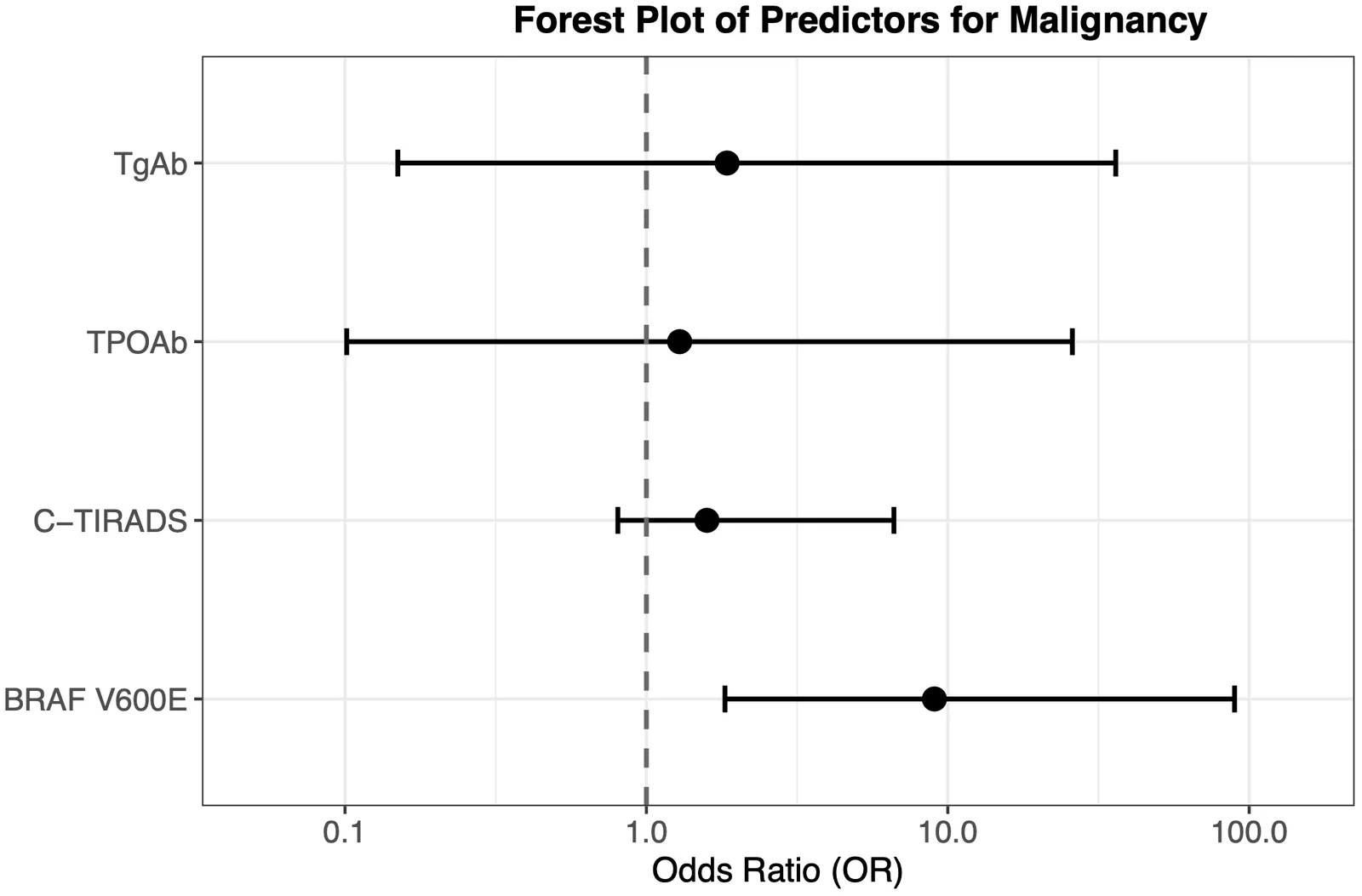
Forest plot of odds ratios (ORs) for relevant variables. The circles and horizontal lines represent the ORs and their 95% confidence intervals (CIs), respectively. The results demonstrate that only the *BRAF* mutation is statistically significant (OR = 9.027; 95% CI: 1.823–89.446; *P* = 0.005), whereas the CIs of all the other variables cross the null line (OR = 1.0).

### 3.4 Receiver operating characteristic curve analysis

ROC curve analysis was performed to evaluate and compare the predictive performance of individual diagnostic markers and the multiparametric combined model. The predictive value (AUC) of individual parameters varied considerably. Immunological markers showed limited diagnostic utility, with AUCs of 0.546 and 0.563 for TgAb and TPOAb, respectively. C-TIRADS demonstrated a moderate predictive performance, with an area under the curve (AUC) of 0.673. Among single markers, the BRAF V600E mutation exhibited the highest diagnostic efficacy, with an AUC of 0. 736. To further optimize diagnostic accuracy, a combined model integrating all individual parameters was constructed. This combined model significantly outperformed all the single indicators, achieving an excellent AUC of 0.821 (**Figure 3**). This substantial increase in the area under the curve indicates that integrating molecular markers (BRAF V600E), serological indicators (TgAb, TPOAb), and imaging features (C-TIRADS) yields the most robust and accurate prediction of malignancy.

**Figure 3:**
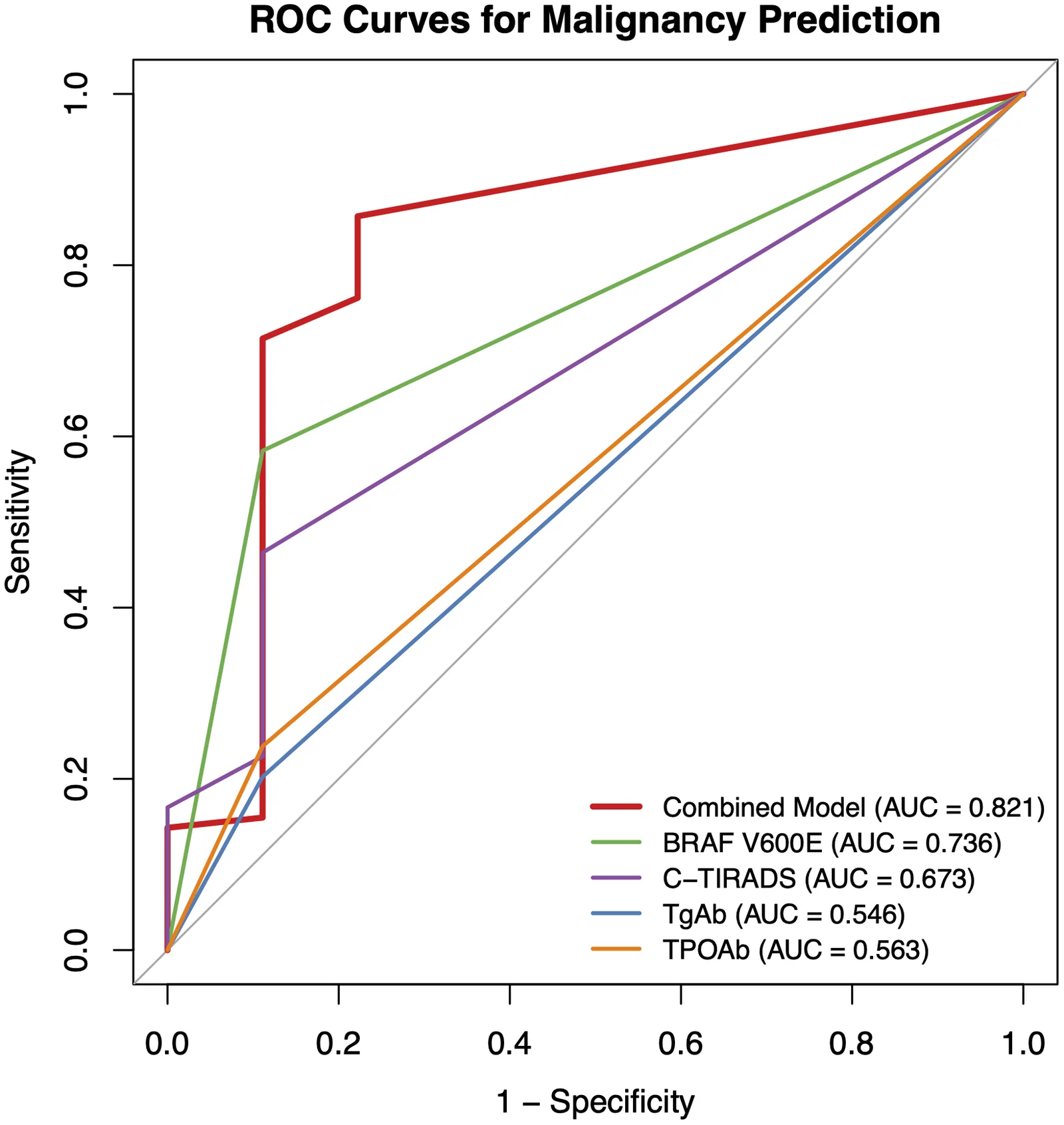
Receiver operating characteristic (ROC) curves. The plot compares the predictive performance of individual biomarkers with that of the multiparameter combined model for malignancy. Compared with the individual predictors, the combined model (red line, AUC = 0.821) achieved the highest discriminative performance: BRAF V600E (green line, AUC = 0.736), C-TIRADS (purple line, AUC = 0.673), TPOAb (orange line, AUC = 0.563), and TgAb (blue line, AUC = 0.546). The diagonal gray dashed line represents the line of no discrimination (AUC = 0.5). In terms of the Youden index, the optimal probability cutoff for the combined model was 0.768, with a sensitivity of 85.7% and a specificity of 77.8%.

To evaluate the incremental value of each predictor category, three sequential models were constructed. Model 1, which included BRAF V600E alone, yielded an AUC of 0.736. The addition of the C-TIRADS in Model 2 increased the AUC to 0.806 (ΔAUC=0.070; DeLong P = 0.062). Further incorporation of TgAb and TPOAb in Model 3 resulted in a modest increase in the AUC to 0.821 (ΔAUC=0.015, DeLong P = 0.548) (**Supplementary Table 1**). Compared with BRAF V600E alone, the final multimodal model had a significantly greater AUC (0.821 vs. 0.736; DeLong P = 0.041) (**Supplementary Table 2**).

### 3.5 Nomogram development

On the basis of the multivariable predictive model generated by the Firth logistic regression, a visual nomogram was constructed to facilitate individualized evaluation of malignancy risk in Bethesda III nodules (**Figure 4**). The nomogram quantitatively integrates the contribution of each predictor by assigning corresponding points to the TgAb status, TPOAb status, C-TIRADS category, and BRAF V600E mutation status. Among these variables, BRAF V600E mutation contributed the greatest weight to the total score (82 points), followed by the C-TIRADS classification (up to 100 points), while TgAb and TPOAb positivity contributed 28 and 14 points, respectively. Clinicians can calculate the total score by summing the points assigned to each predictor and then convert the total points into an individualized predicted probability of malignancy using the probability scale at the bottom of the nomogram.

**Figure 4:**
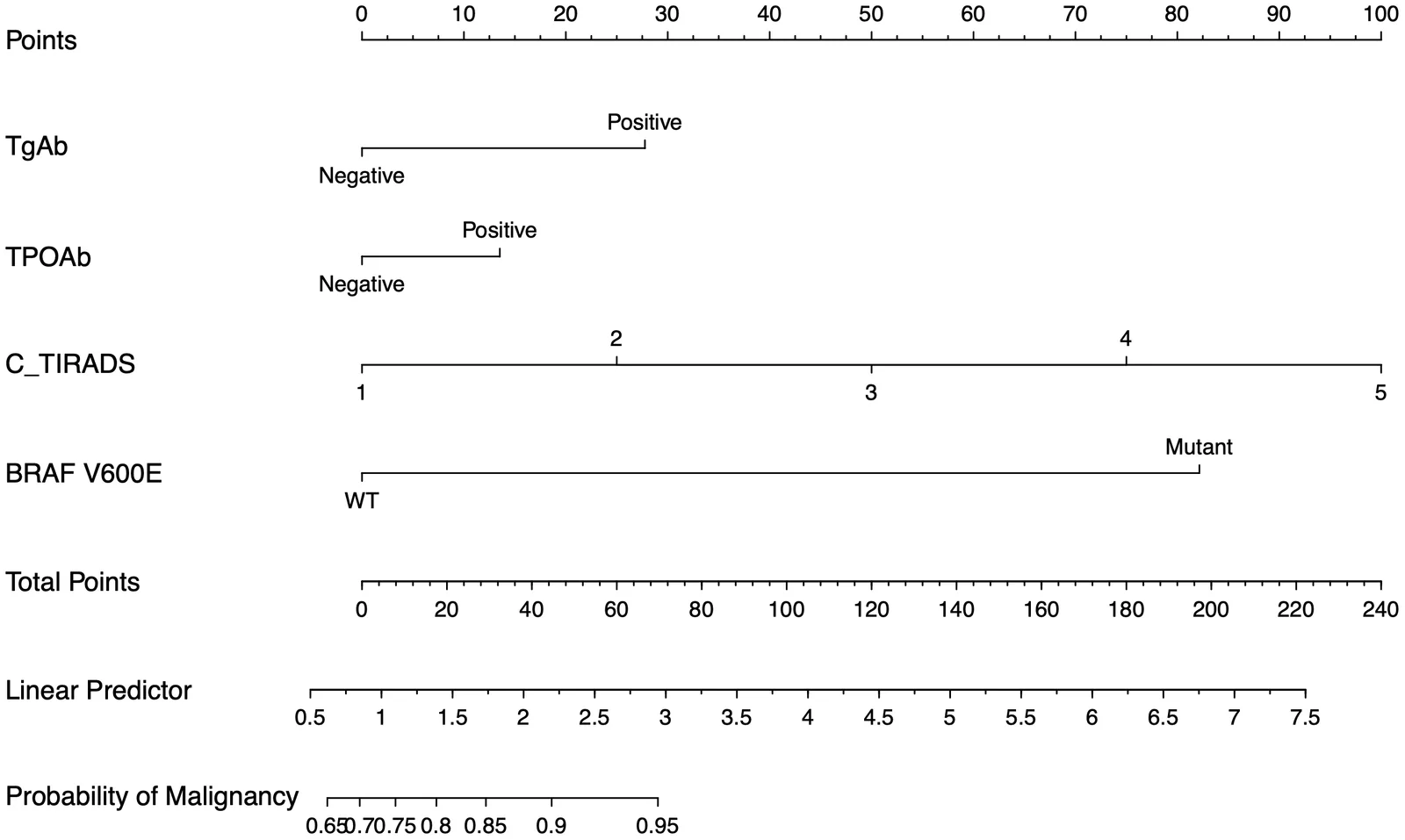
Nomogram for predicting the risk of malignancy. The model integrates TgAb, TPOAb, C-TIRADS, and BRAF V600E mutation status, allowing estimation of individual malignancy probabilities by calculating the total score. The total points were obtained by summing the points assigned to each predictor. According to the results of the ROC curve analysis, a total score of approximately 12 points corresponded to the optimal malignancy probability threshold (0.768), and patients with a total score ≥12 were considered to have an increased predicted risk of malignancy.

ROC curve analysis, based on the Youden index, was performed to determine the optimal probability threshold for malignancy prediction. The optimal predicted probability cutoff was 0.768, with a sensitivity of 85.7% and a specificity of 77.8%. According to the nomogram scale, this threshold corresponded to approximately 12 total points. Therefore, patients with a total nomogram score ≥12 were considered to have an increased predicted risk of malignancy. The nomogram provided individualized risk estimates across a broad probability range, allowing clinicians to perform quantitative risk stratification for Bethesda III thyroid nodules.

### 3.6 Calibration curve

Internal validation of the constructed Firth logistic multimodal regression model was executed using bootstrap resampling (1000 iterations), and a calibration curve was generated (**Figure 5**). The model yielded a C-index (equivalent to the AUC) of 0.821, reflecting excellent discriminative ability. The calibration curve demonstrated a highly favorable overall fitting trend between the nonparametric curve (connected by scattered triangles) and the diagonal line, and the curve indicated the ideal performance. Specifically, in the high-predicted-probability interval (>0.70), the malignancy risk predicted by the model was highly concordant with the actual malignant proportions observed in surgical histopathology. The Brier score was calculated at 0.073, and the goodness-of-fit test revealed no statistically significant variance (S: p = 0.360 > 0.05).

**Figure 5:**
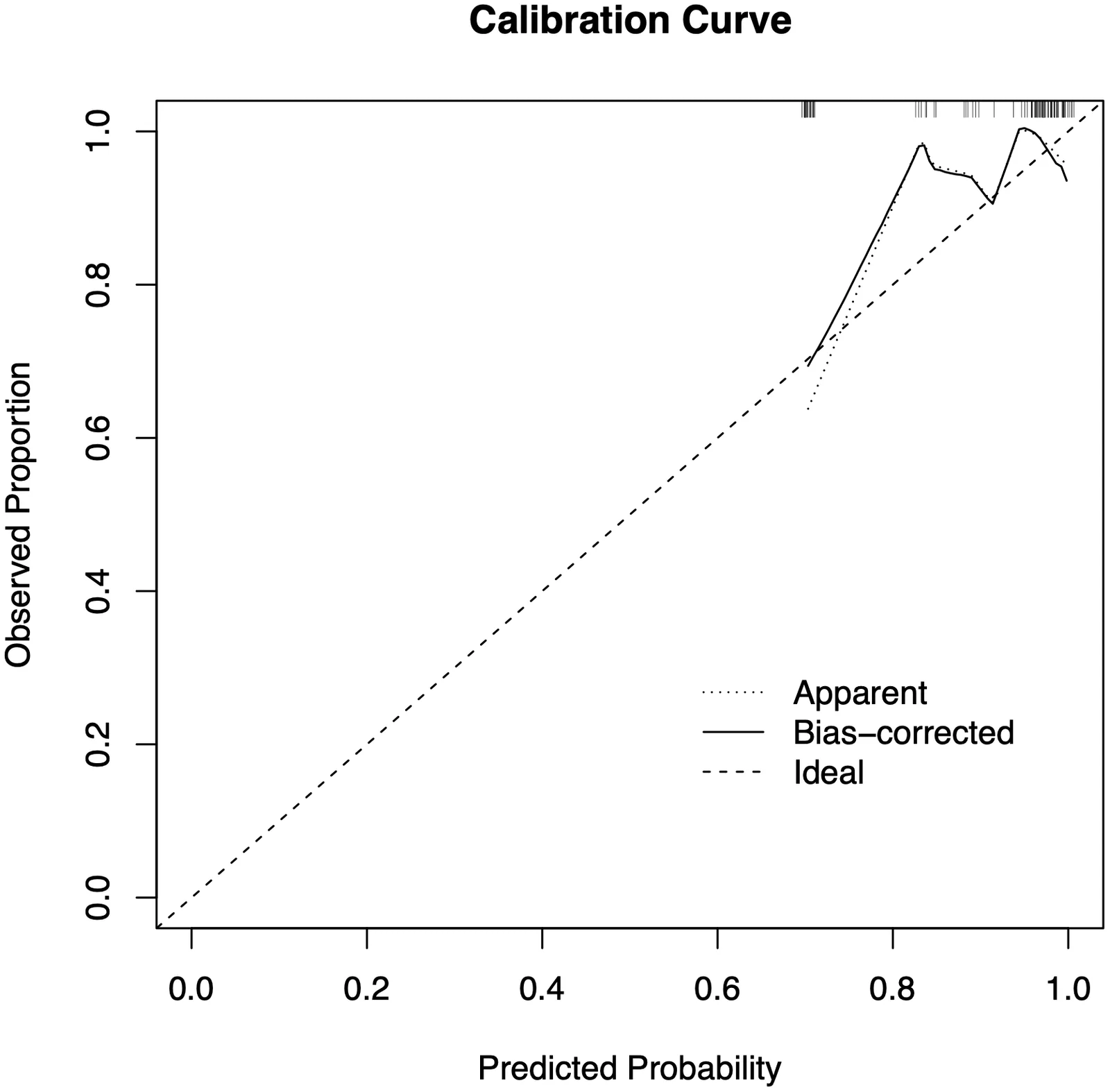
Calibration plot of the Firth logistic regression model for predicting malignancy risk. The solid black line closely aligns with the diagonal ideal reference line, indicating high consistency between the predicted risk of malignancy and the actual clinical incidence (C-index = 0.821).

### 3.7 Decision curve analysis

DCA was employed to assess the clinical applicability of the predictive models, and the results are illustrated in **Figure 6**. Within the threshold probability range of 0% to 65%, the net benefit curves for the combined model, BRAF V600E model, C-TIRADS model, and serological model largely overlapped, indicating comparable clinical benefits within this interval.

**Figure 6:**
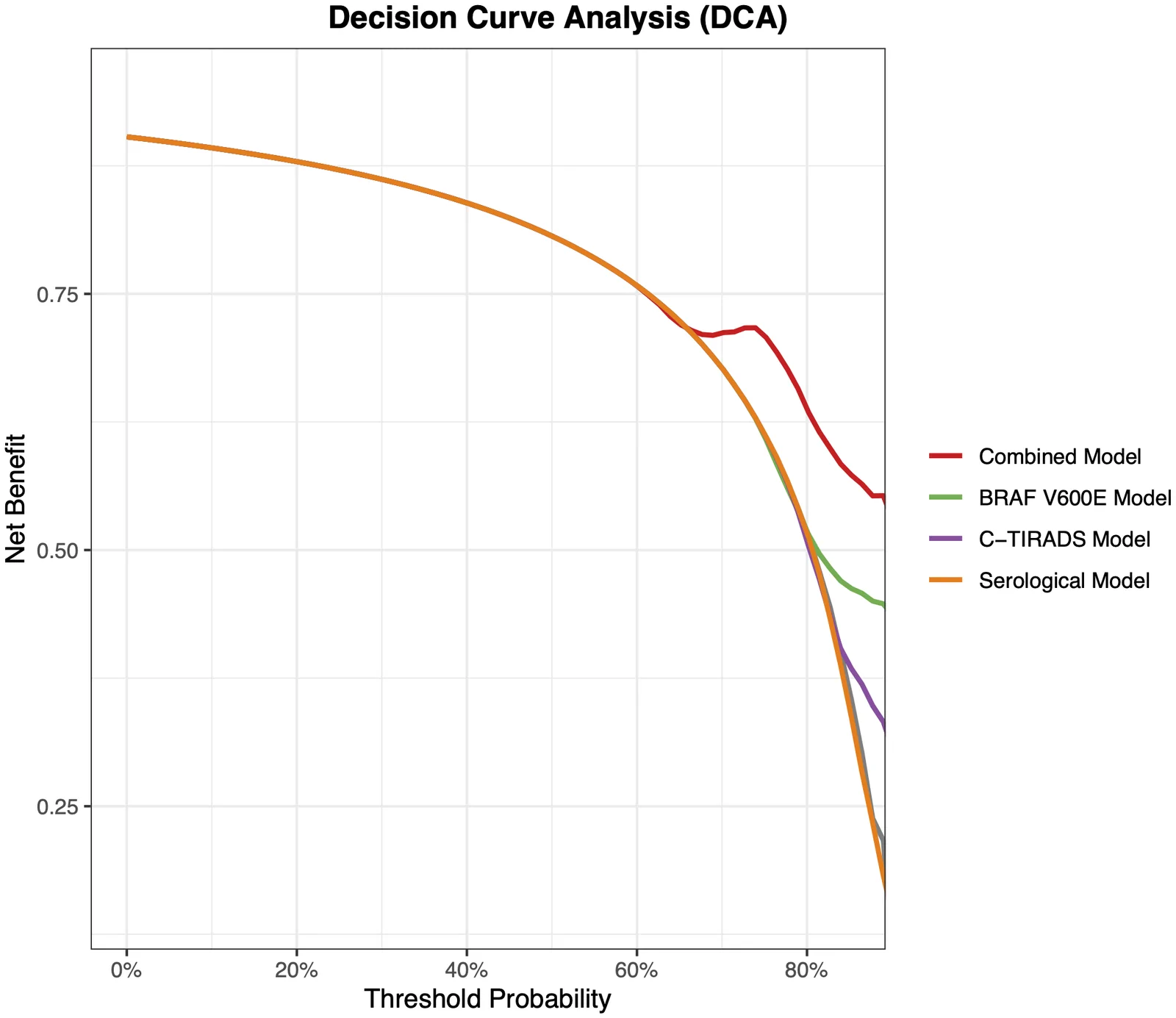
Decision curve analysis (DCA) of the four diagnostic models for thyroid malignancy. The X-axis represents the threshold probability, and the Y-axis represents the clinical net benefit. The colored lines represent the four prediction models: the combined model (red), the *BRAF* V600E model (green), the C-TIRADS model (purple), and the serological model (orange). When the threshold probability exceeds 65%, the curve of the combined model remains consistently above that of the individual models, indicating that it provides the greatest clinical net benefit and optimal clinical utility across a wide range of risk thresholds.

However, when the threshold probability exceeded 65%, distinct divergences in net benefits emerged across the models. The combined model consistently sustained the highest level of net benefit across the mid-to-high threshold probability ranges, showing a relatively gradual decline and indicating optimal clinical decision-making efficacy. Among the univariable models, the BRAF V600E model surpassed both the C-TIRADS and serological models, whereas the serological model demonstrated the lowest net benefit in the high-threshold probability interval.

These findings suggest that compared with the individual use of serological markers, ultrasound features, or BRAF V600E mutation status, the combined multimodal model provides greater net clinical benefit over a broader spectrum of threshold probabilities, underscoring its superior application value in preoperative risk evaluation and clinical decision support for Bethesda III thyroid nodules.

## 4 Discussion

Owing to significant cytological heterogeneity, Bethesda Category III thyroid nodules are widely acknowledged as “gray zones” in the diagnosis and management of thyroid lesions. While the overall malignancy risk reported by the Bethesda system range from 5% to 30% (28, 29), substantial variations exist across different clinical centers and patient selection criteria, leading to a lack of consensus on optimal management strategies. Striking a balance between avoiding unnecessary surgical procedures and mitigating the risk of missed malignant diagnoses remains the core challenge in the precise management of these nodules. In this study, we addressed this challenge by developing a multimodal predictive model that integrates serological indicators, ultrasound risk stratification, and molecular diagnostics and employing Firth logistic regression to ensure robust modeling. Our analysis revealed that the BRAF V600E mutation was the only statistically significant independent risk factor. Furthermore, compared with any single modality, the combined model demonstrated superior overall diagnostic efficacy, underscoring the substantial potential value of multidimensional data integration in evaluating the risk of Bethesda III nodules.

The most pivotal finding of this study is the strong predictive ability of the BRAF V600E mutation, which was maintained even in the multivariable analysis (OR = 9.03, 95% CI: 1.82–89.45; P = 0.005). The BRAF V600E mutation represents the most frequent driver gene alteration in papillary thyroid carcinoma. It facilitates tumor cell proliferation, invasion, and immune evasion via continuous activation of the MAPK signaling pathway, making it a critical molecular event in thyroid carcinogenesis (15). Prior research has established that BRAF V600E testing is highly specific for cytologically indeterminate nodules, significantly enhancing the identification of malignant lesions (16, 17). BRAF V600E mutation testing represents an early and widely investigated molecular approach for thyroid cancer risk assessment. However, as a single-gene assay, the diagnostic performance of BRAF V600E testing is limited by the genetic heterogeneity of thyroid cancer and the absence of BRAF V600E alterations in a proportion of malignant nodules. In recent years, molecular diagnostics have evolved towards more comprehensive strategies, including multigene mutation panels and gene expression classifiers. Commercial platforms such as the Afirma Genomic Sequencing Classifier (GSC) and ThyroSeq v3 have demonstrated improved risk stratification performance for indeterminate thyroid nodules and have shown the potential to reduce unnecessary surgeries (18–21).

Nevertheless, the clinical implementation of these advanced molecular platforms remains influenced by factors such as cost, availability of sequencing infrastructure, and differences in health care systems. Therefore, BRAF V600E testing, particularly when it is integrated with ultrasound characteristics and clinical parameters, may still provide practical value in routine clinical settings where comprehensive molecular testing is not readily accessible. Our findings suggest that combining BRAF V600E with the C-TIRADS and thyroid-related clinical indicators improve the preoperative risk assessment of Bethesda III nodules. Future studies should incorporate emerging molecular technologies, including multigene panels and transcriptomic classifiers, to further optimize prediction models and evaluate whether the integration of traditional biomarkers with advanced molecular profiles can provide more accurate and individualized risk stratification for indeterminate thyroid nodules. Our results closely align with these reports, reinforcing that even within the highly heterogeneous Bethesda III population, BRAF V600E remains one of the most clinically valuable molecular biomarkers. Notably, the area under the curve (AUC) for isolated BRAF V600E testing reached 0.736, the highest among all single indicators, indicating strong discriminatory power for malignancy risk. From a practical clinical perspective, while next-generation sequencing (NGS) platforms such as ThyroSeq have shown enhanced diagnostic accuracy in recent years (22), their high costs and limited availability in primary care hospitals restrict widespread use. In contrast, BRAF V600E testing offers significant advantages in terms of operational simplicity, cost-effectiveness, and reproducibility, making it highly suitable for implementation in primary healthcare settings.

Despite the profound predictive strength of the BRAF V600E mutation, a single parameter is insufficient to comprehensively capture the complex biological characteristics of Bethesda III nodules. The combined multimodal model constructed in this study achieved an AUC of 0.821, outperforming all single indicators, suggesting that the fusion of multidimensional information can substantially increase risk assessment capabilities. Recent investigations into Bethesda III nodules have progressively shifted from single-factor evaluations toward comprehensive predictive modeling. For example, Zhang et al. developed a predictive model that combines ultrasound and cytology (23), whereas Li et al (24). utilized machine learning methodologies to integrate multidimensional clinical features. Both approaches demonstrated that combined models deliver superior predictive performance. Our findings are consistent with these trends and robustly support the philosophy of multidimensional risk evaluation. While TgAb, TPOAb, and C-TIRADS did not reach statistical significance in the multivariable analysis, their inclusion in the model increased overall predictive efficacy. This implies that variables lacking independent risk factor status may still enhance a model’s discriminative power by providing complementary information. Biologically, the C-TIRADS reflects morphological characteristics, TgAb and TPOAb partially mirror the host immune status and chronic inflammatory milieu (25), and BRAF V600E indicates tumor-driving genomic alterations. Therefore, these distinct dimensions possess inherent informational complementarity.

A key methodological strength of this study was the deployment of Firth’s penalized likelihood logistic regression rather than conventional logistic regression for model construction. The study cohort exhibited profound class imbalance, with 90.32% malignant cases and only 9 benign cases. Under such conditions, traditional maximum likelihood estimation is prone to parameter bias and the problem of complete separation, resulting in unstable models (26). Firth logistic regression effectively mitigated the small-sample bias through penalized likelihood estimation, yielding highly robust parameter estimates, and it is widely regarded as an essential technique for handling rare events and imbalanced datasets (27). By further combining ROC curves, bootstrap internal validation, calibration curves, and DCA, we comprehensively evaluated the model. The results confirmed excellent discrimination, consistency, and clinical net benefit, demonstrating the high suitability of the Firth method for small-sample Bethesda III investigations.

It is vital to explicitly contextualize the exceptionally high malignancy rate (90.32%) observed in this study, which significantly exceeds standard reports for the Bethesda III category. This deviation is primarily attributable to selection bias, as the study population exclusively included patients who underwent surgical resection, resulting in a surgically enriched cohort. In the real-world clinical practice, Bethesda III patients who proceed to surgery frequently harbor concurrent high-risk sonographic features, test positive for BRAF V600E, or express a strong personal preference for surgical intervention. Consequently, the proportion of malignancies in this subgroup is inherently higher than that in the general Bethesda III population. While this selection bias enhances the model’s ability to identify high-risk individuals, it may also lead to an overestimation of predicted probabilities, thereby limiting the generalizability of the findings to an unselected Bethesda III screening population. Thus, our proposed model is best utilized for Bethesda III patients who have already entered the surgical decision-making phase or who exhibit elevated baseline risk, rather than functioning as a universal primary screening tool.

## 5 Limitations of the study

This study has several limitations. First, as a single-center retrospective analysis, inherent selection bias is unavoidable. Second, the sample size is relatively limited, particularly concerning the low number of benign cases, which may impact the stability of effect estimations for certain variables. Although Firth penalized logistic regression was applied to reduce small-sample bias and internal validation was performed using bootstrap resampling, external validation in larger, independent cohorts is still necessary before clinical application of the proposed model. Third, the molecular scope was confined to BRAF V600E testing. We did not integrate other molecular indicators, such as RAS mutations, TERT promoter mutations, RET/PTC fusions, or broad NGS panels. Finally, the study relied strictly on bootstrap internal validation and lacked an independent external validation cohort. Future multicenter, large-scale, prospective studies that incorporate a broader array of molecular markers and advanced artificial intelligence algorithms are warranted to further optimize the model’s performance and clinical applicability.

## 6 Conclusion

This study demonstrated that the BRAF V600E mutation is an independent predictor of malignancy in Bethesda Category III thyroid nodules. TgAb and TPOAb did not demonstrate strong independent discrimination when evaluated individually. However, autoimmune serological markers were included as complementary variables within the multimodal framework, aiming to capture additional biological information that may not be reflected by ultrasound or molecular testing alone. To minimize potential confounding caused by autoimmune-related antibody elevation and inflammatory changes on ultrasound, patients with Hashimoto thyroiditis were excluded from this study. Nevertheless, we acknowledge that these exclusion criteria may limit the generalizability of our findings to patients with concomitant autoimmune thyroid disease. A multimodal prediction model that integrates TgAb, TPOAb, C-TIRADS, and BRAF V600E exhibits excellent diagnostic efficacy and high clinical net benefit. For high-risk Bethesda III patients undergoing surgical evaluation, this model holds significant promise in providing crucial adjunctive support for preoperative risk stratification and personalized clinical decision-making. Our findings are hypothesis-generating and require further validation in larger, prospective, multicenter cohorts before clinical implementation. Future studies incorporating independent external validation cohorts and more diverse patient populations will be necessary to confirm the robustness and generalizability of the proposed model.

## Data Availability

The original contributions presented in the study are included in the article/**Supplementary Material**. Further inquiries can be directed to the corresponding author.
