# Supplementary material for: A malignancy risk prediction model for Bethesda category III thyroid nodules based on serological indicators, C-TIRADS, and BRAF V600E testing
Source: Front Endocrinol (Lausanne). 2026 Sep 24;17:1919450. doi: 10.3389/fendo.2026.1919450 (PMC13645610; doi:10.3389/fendo.2026.1919450)
Supplement: Supplementary file 1 [file DataSheet2.pdf]

0. 加载核心分析与导出包

==

library(tidyverse) # 数据清洗与ggplot2绘图 library(logistf) # Firth惩罚似然Logistic回归 library(pROC) # ROC曲线绘制与AUC计算 library(rms) # 列线图(Nomogram)与校准曲线(Calibration) library(dcurves) # 决策曲线分析(DCA) library(gtsummary) # 自动化临床基线表生成 library(gridExtra) # 【新增】核心包：用于将数据表格完美排版并导出为PDF

自动创建目标输出文件夹（如果不存在的话）

if(!dir.exists("project")) { dir.create("project") }

==

1. 数据读取与多变量并行预处理

==

data <- read.csv("thyroid_data.csv", check.names = FALSE)

规范化特殊字符表头，防止底层建模语法解析报错

colnames(data)[colnames(data) == "C-TIRADS"] <- "C_TIRADS" colnames(data)[colnames(data) == "BRAF V600E"] <- "BRAF_V600E"

显式保留数值型 0/1 变量，专供无误差的二分类混淆矩阵及Firth模型计算

data$Malignant_num < -data$Malignant data$TgAb_num < -data$TgAb data $TPOAb_num < -data$TPOAb data$BRAF_num < -data$BRAF_V600E data $C_TIRADS < -as.numeric(data$C_TIRADS)

同时生成英文 Factor 标签，专供 Table 1 和列线图绘制

data$Malignant_factor < -factor(data$Malignant, levels = c(0, 1), labels = c("Benign", "Malignant")) data$TgAb_factor < -factor(data$TgAb, levels = c(0, 1), labels = c("Negative", "Positive")) data$TPOAb_factor < -factor(data$TPOAb, levels = c(0, 1), labels = c("Negative", "Positive")) data$BRAF_factor < -factor(data$BRAF_V600E, levels = c(0, 1), labels = c("WT", "Mutant"))

==

2.2 临床特征基线表 (对应 表1) -> 导出 PDF

==

table1 <- data %>% select(TgAb, TPOAb, C_TIRADS, BRAF_V600E, Malignant_factor) %>% tbl_summary(by = Malignant_factor, statistic = list(all_categorical() ~ "{n}", all_continuous() ~ "{median} ({p25}, {p75})"), label = list(TgAb ~ "TgAb", TPOAb ~ "TPOAb", C_TIRADS ~ "C-TIRADS", BRAF_V600E ~ "BRAF V600E")) %>% add_overall() %>% modify_header(label = "**Variables**") %>% bold_labels()

在 RStudio 中输入后可在右下角 Viewer 窗格直接查看与导出网页格式表格

table1

===

2.3 Firth 多因素回归分析与森林图 (对应 表4 & 图2) -> 导出 PDF

===

拟合回归模型

fit_firth <- logistf(Malignant_num ~ TgAb_num + TPOAb_num + C_TIRADS + BRAF_num, data = data)

提取回归参数、OR 值及 95% CI

firth_summary <- data.frame(Variable = c("TgAb", "TPOAb", "C-TIRADS", "BRAF V600E"), OR = exp(coef(fit_firth))[-1], Lower_CI = exp(confint(fit_firth))[-1, 1], Upper_CI = exp(confint(fit_firth))[-1, 2], P_value = fit_firth$prob[-1])

格式化多因素表格并导出为 PDF

firth_summary_formatted <- firth_summary %>% mutate(across(where(is.numeric), ~ round(., 3))) pdf("project/Table4_Firth_Regression_Results.pdf", width = 7, height = 2.5) grid.table(firth_summary_formatted, rows = NULL) dev.off() print("✔ Table 4 (Firth回归回归表) 已成功导出至 project/Table4_Firth_Regression_Results.pdf")

绘制并自动保存 图2：森林图

firth_summary$Variable <- factor(firth_summary$Variable, levels = rev(firth_summary$Variable)) forest_plot <- ggplot(firth_summary, aes(x = OR, y = Variable)) + geom_point(size = 3.5, color = "black") + geom_errorbarh(aes(xmin = Lower_CI, xmax = Upper_CI), height = 0.15, linewidth = 0.8, color = "black") + geom_vline(xintercept = 1, linetype = "dashed", color = "grey40", linewidth = 0.8) + scale_x_log10(breaks = c(0.1, 1, 10, 100), limits = c(0.05, 150)) + theme_bw() + labs(x = "Odds Ratio (OR)", y = "", title = "Figure 2: Forest Plot of Predictors for Malignancy") + theme(axis.text = element_text(size = 11), axis.title = element_text(size = 12), plot.title = element_text(hjust = 0.5, face = "bold"))

ggsave("project/Figure2_Forest_Plot.pdf", plot = forest_plot, width = 7, height = 4.5) print("✔ Figure 2 (森林图) 已成功导出至 project/Figure2_Forest_Plot.pdf")

===

自动拦截设备并保存多指标叠加的 ROC 曲线

pdf("project/Figure3_ROC_Curves.pdf", width = 6, height = 6)

1. 先画联合模型（作为主图底层）

plot(roc_combined, col = "#E41A1C", lwd = 3, legacy.axes = TRUE, main = "ROC Curves for Malignancy Prediction", xlab = "1 - Specificity", ylab = "Sensitivity")

2. 依次叠加另外 4 个单项指标

plot(roc_braf, col = "#4DAF4A", lwd = 2, add = TRUE) plot(roc_tirads, col = "#984EA3", lwd = 2, add = TRUE) plot(roc_tg, col = "#377EB8", lwd = 2, add = TRUE) # TgAb 蓝色线 plot(roc_tpoab, col = "#FF7F00", lwd = 2, add = TRUE) # TPOAb 橙色线

3. 更新图例（使用 $auc 属性直接读取，完美避开报错）

legend("bottomright", legend = c(sprintf("Combined Model (AUC = %.3f)", roc_combined $auc), sprintf("BRAFV600E(AUC =$auc), sprintf("C-TIRADS (AUC = %.3f)", roc_tirads $auc), sprintf("TgAb(AUC =$auc), sprintf("TPOAb (AUC = %.3f)", roc_tpoab$auc)), col = c("#E41A1C", "#4DAF4A", "#984EA3", "#377EB8", "#FF7F00"), lwd = c(3, 2, 2, 2, 2), bty = "n", cex = 0.8)

dev.off() print("✔ Figure 3 (5项指标完整版ROC) 已成功修复并导出！")

===

2.5 列线图构建 (对应 图3) -> 导出 PDF

===

1. 首先加载 rms 包（确保后续函数能被找到）

library(rms)

1. 直接修改原始数据集（data）中变量的 label 属性

这是 rms 包最标准的打标签方式

label(data$TgAb_factor) <- "TgAb" label(data$TPOAb_factor) <- "TPOAb" label(data$BRAF_factor) <- "BRAF V600E"

2. 核心步骤：重新打包数据环境（这样新的标签才会被 rms 记住）

dd <- datadist(data) options(datadist = "dd")

3. 重新拟合模型（模型会自动继承上面设置的标准标签）

fit_lrm <- lrm(Malignant_num ~ TgAb_factor + TPOAb_factor + C_TIRADS + BRAF_factor, data = data, x = TRUE, y = TRUE, penalty = 0.1)

4. 构建列线图

nom <- nomogram(fit_lrm, fun = plogis, funlabel = "Probability of Malignancy")

5. 导出完美的列线图 PDF

pdf("project/Figure4_Nomogram.pdf", width = 10, height = 7) plot(nom) dev.off()

print("✔ 标签已成功应用！请查看更新后的 project/Figure4_Nomogram.pdf")

===

2.7 内部验证校准曲线 (对应 图4) -> 导出 PDF

===

cal <- calibrate(fit_lrm, method = "boot", B = 1000)

拦截输出并保存校准曲线

pdf("project/Figure5_Calibration_Curve.pdf", width = 6, height = 6) plot(cal, xlim = c(0, 1), ylim = c(0, 1), xlab = "Predicted Probability", ylab = "Observed Proportion", main = "Figure 4: Calibration Curve", subtitles = FALSE) dev.off() print("✔ Figure 5 (校准曲线 Calibration) 已成功导出至 project/Figure5_Calibration_Curve.pdf")

==

2.8 临床决策曲线分析 DCA (对应 图5) -> 导出 PDF

==

data$pred_{b}raf_{o}nly < -glm(Malignant_{n}um~BRAF_{n}um, data = data, family = binomial)$fitted.values data$pred_{t}irads_{o}nly < -glm(Malignant_{n}um~C_{T}IRADS, data = data, family = binomial)$fitted.values data$pred_{s}erology < -glm(Malignant_{n}um~TgAb_{n}um + TPOAb_{n}um, data = data, family = binomial)$fitted.values

dca_plot <- dca(Malignant_num ~ pred_prob + pred_braf_only + pred_tirads_only + pred_serology, data = data, thresholds = seq(0, 0.99, by = 0.01), label = list(pred_prob = "Combined Model", pred_braf_only = "BRAF V600E Model", pred_tirads_only = "C-TIRADS Model", pred_serology = "Serological Model")) %>% plot(smooth = TRUE) + coord_cartesian(xlim = c(0, 0.85), ylim = c(0.15, 0.95)) + scale_color_manual(values = c("Combined Model" = "#E41A1C", "BRAF V600E Model" = "#4DAF4A", "C-TIRADS Model" = "#984EA3", "Serological Model" = "#FF7F00")) + theme_bw() + labs(title = "Figure 5: Decision Curve Analysis (DCA)", x = "Threshold Probability", y = "Net Benefit") + theme(plot.title = element_text(hjust = 0.5, face = "bold"), legend.position = "right")

ggsave("project/Figure6_DCA_Plot.pdf", plot = dca_plot, width = 7, height = 6) print("✔ Figure 6 (决策曲线 DCA图) 已成功导出至 project/Figure6_DCA_Plot.pdf")

```
print("=========================================================================
==========")
```
